# Fabrication of sharp silicon hollow microneedles by deep-reactive ion etching towards minimally invasive diagnostics

**DOI:** 10.1038/s41378-019-0077-y

**Published:** 2019-08-26

**Authors:** Yan Li, Hang Zhang, Ruifeng Yang, Yohan Laffitte, Ulises Schmill, Wenhan Hu, Moufeed Kaddoura, Eric J. M. Blondeel, Bo Cui

**Affiliations:** 1 Department of Electrical and Computer Engineering, University of Waterloo, 200 University Avenue West, Waterloo, ON N2L 3G1 Canada; 2 ExVivo Labs Inc., 3 Regina Street North, Waterloo, ON N2J 2Z7 Canada

**Keywords:** Electrical and electronic engineering, Microfluidics

## Abstract

Microneedle technologies have the potential for expanding the capabilities of wearable health monitoring from physiology to biochemistry. This paper presents the fabrication of silicon hollow microneedles by a deep-reactive ion etching (DRIE) process, with the aim of exploring the feasibility of microneedle-based in-vivo monitoring of biomarkers in skin fluid. Such devices shall have the ability to allow the sensing elements to be integrated either within the needle borehole or on the backside of the device, relying on capillary filling of the borehole with dermal interstitial fluid (ISF) for transporting clinically relevant biomarkers to the sensor sites. The modified DRIE process was utilized for the anisotropic etching of circular holes with diameters as small as 30 μm to a depth of >300 μm by enhancing ion bombardment to efficiently remove the fluorocarbon passivation polymer. Afterward, isotropic wet and/or dry etching was utilized to sharpen the needle due to faster etching at the pillar top, achieving tip radii as small as 5 μm. Such sharp microneedles have been demonstrated to be sufficiently robust to penetrate porcine skin without needing any aids such as an impact-insertion applicator, with the needles remaining mechanically intact after repetitive penetrations. The capillary filling of DRIE-etched through-wafer holes with water has also been demonstrated, showing the feasibility of use to transport the analyte to the target sites.

## Health monitoring: microneedle fabrication

Microneedles are fabricated with a diameter as small as 30 µm and a tip radius of 5 µm, enabling skin penetration for in-vivo sampling of interstitial fluid. A key aspect to wearable health monitoring is the availability of devices that can sample key biomarkers in-vivo; microneedles are particularly promising for minimally invasive sampling of interstitial fluid. However, careful control of the microneedle size, geometry and quality are needed for effective penetration and sampling. Now, a team led by Bo Cui from University of Waterloo, and Eric Blondeel from ExVivo Labs Inc., demonstrates a modified deep reactive ion etching process for creating cone-shaped silicon microneedles with a hole opening for fluid extraction. Capillary filling of the holes is demonstrated, as well as repeatable penetration of porcine skin.

## Introduction

Wearable healthcare monitoring technologies have the potential for dramatically expanding the capability of data acquisition regarding an individual’s health^1,2^. Recently, microneedle technologies have become increasingly interesting for unleashing the potential to minimally invasively interfere with an individual’s biochemistry, such as for drug delivery^3,4^, interstitial fluid sampling^5,6^, and diagnostics^7,8^. Microneedles require only a small area of skin to be penetrated at a limited depth, resulting in minimal irritation of the dermal layers associated with pain and tissue damage.

Silicon microneedles are desirable due to their excellent biocompatibility and, in particular, mechanical properties superior to those of polymer and metal, such as a nonductile nature, high Young’s Modulus, and indentation hardness enabling skin penetration without breakage in the skin^9^. Despite some concerns, silicon material revealed biocompatibility in a baseline battery of ISO 10993 physicochemical and biocompatibility tests^10^. With silicon implantation, comprehensive studies of the immunohistochemistry of brain tissues demonstrated that silicon devices and the byproducts of their dissolution in the intracranial space are biocompatible^11,12^. In particular, the Food and Drug Administration (FDA) has granted clearance for silicon devices, such as silicon microneedles (NanoPass Technologies Ltd., https://www.nanopass.com/)^13^ and silicon Utah array electrodes (Blackrock Microsystems LLC, https://blackrockmicro.com/electrode-main/)^14^. In comparison with other microneedle materials, e.g., polymers^15^ and metals^16^, silicon has the advantages of being compatible with well-established micro/nanofabrication technologies for enabling added functionalities, such as through the monolithic integration of microneedles and complementary metal-oxide-semiconductor (CMOS) circuitry for continuous and real-time diagnostics^17–20^.

Interstitial fluid (ISF) holds great promise as an alternative source of biomarkers for blood plasma^21,22^. ISF, formed by blood transcapillary filtration, has a composition comparable to that of plasma, indicating significant untapped potential for a wide range of diagnostics. Proteomic and metabolomics analysis indicates that ISF is highly similar to both plasma and serum^21,22^. It has also been shown that certain biomarkers (e.g., glucose) in ISF at equilibrium have concentration levels that correlate well with those in clinically relevant blood plasma^23,24^. By withdrawing ISF, biomarkers of clinical interest are measured either off-line by standard commercial methods^22^ or in-line by integrated biosensors^7^. In the latter configuration, ISF is transported through the needle lumen to the biosensor integrated on the backside of the device^7,23^. Both methods necessitate a large amount of ISF volume to improve the diagnostic consistency. The epidermis largely comprises keratinocytes cells, and ISF-filled compartments are sparsely distributed in the upper region of the dermis (papillary), enveloped by the structural molecules of the interstitium matrix such as collagen frameworks^24–26^. The sampling of ISF using microneedles involves penetration through a deformable, elastic skin barrier, which is a challenge often resulting in incomplete needle penetration^27,28^. The inhomogeneity of ISF population results in inconsistent recovery and limited volume (typically submicroliter scale), and enhanced recovery necessitates fluid extraction mechanisms such as vacuum suction^5^. In addition, millimeter-long needles have enabled the recovery of ISF at up to tens of microliters from the deeper dermal region (i.e., the reticular dermis)^22^. However, ISF interrogation in the reticular dermis likely results in inevitable contact between the needle and sensory nerve endings, as well as blood capillaries.

On the other hand, bringing the sensor closer to the microneedle tip enables consistent in-vivo monitoring of biomarkers of clinical interest with reduced ISF volumes such as subnanoliter amounts^8,16,29–31^. The close proximity between the sensor and interstitium (containing ISF) ensures attaining an equilibrium protein concentration, as the biomarkers of clinical interest surrounding the sensor have a good correlation with the free ISF (and plasma)^32^. Such sensor configuration also enables reducing the lag time in ISF corresponding to changes of the blood glucose level associated with the capillary-to-sensor diffusion time^30^. The device architecture mainly relies on an assembly process to place the biosensor compartment (i.e., electrodes of electrochemical transducers) inside the borehole of the hollow microneedle. By leveraging CMOS/MEMS technologies (e.g., through-silicon via (TSV) filling of copper or doped poly-silicon), metallization inside the hole for electrode fabrication can therefore be accomplished with wafer-level processes amenable to mass production^33,34^.

The state-of-the-art fabrication process for making silicon hollow microneedles typically relies on deep-reactive ion etching (DRIE) of holes with small diameters from both sides because of the otherwise limited hole etching depth from a single side associated with so-called aspect ratio-dependent etching (ARDE)^35–41^. The challenge in this process is the need of precise double-sided alignment, especially with the ARDE-induced tapering hole profile^41,42^, and the significant hole widening during the needle sharpening process using isotropic wet etching. Alternatively, relatively large through-wafer holes are etched from one side through a combination of tapered and straight profiles^43,44^, whereas a smoothly tapered profile is more desirable^45^.

This paper presents the DRIE of silicon hollow microneedles, which resemble elongated cones with smooth tapering from the shank to extreme sharpness. A triple-phase modified Bosch process was developed to enable the production of sufficiently deep holes with small diameters from a single side of the Si wafer. As such, more processing flexibility was provided for optimal pillar etching without the compromise of simultaneous pillar and hole etching. Afterward, the isotropic etching process was managed to selectively remove the unwanted silicon to open holes and to achieve needle sharpening. This process also prevents the holes from undesirable etching by the aggressive etchant.

## Design of THE microneedle chip

Microneedle-enabled transdermal minimally invasive platforms typically utilize miniaturized needles of several hundred micrometers, resulting in a limited skin penetration depth to minimize the patient’s discomfort. It is highly desirable to interrogate ISF in the superficial papillary dermis, which is directly beneath the epidermis-dermis junction (with a variable depth of 100–200 μm from the skin surface), without triggering the sensory nerve endings and blood capillaries in the deeper dermis (deeper papillary and reticular)^24,46^. Such pain-free ISF interrogation can be enabled by an array of ~150 µm-long microneedles, which penetrate through the thinner epidermis (and stratum corneum) exactly, only touching the ISF-filled compartment. It is noteworthy that the optimization of the needle shape (e.g., via a smaller needle base) can mitigate the incomplete penetration resulting from the so-called bed-nail effects and the viscoelastic nature of skin. Furthermore, fluid sampling approaches are prone to clogging associated with tissue coring at the insertion sites, which necessitates accurate placement of the borehole off-center from the needle axis^44^. This also enables improving the sharpness of the apex (essentially solid, rather than hollow) after the sharpening process.

**Fig. 1:**
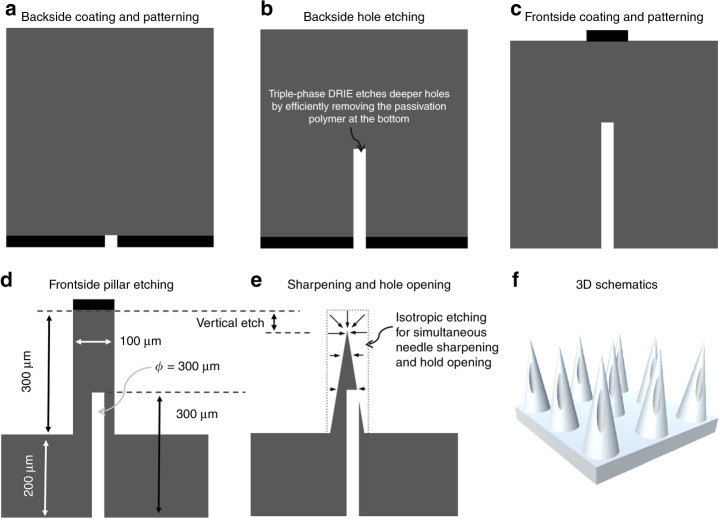
**Schematic fabrication process of silicon hollow microneedle arrays**

Therefore, our microneedle array was designed to have silicon pillars of 200–300 μm long to achieve the needle length of ~150 µm for etching vertically, along with lateral sharpening (see details in Fig. 1). Second, the diameter of the pillars was chosen as 100 µm to provide sufficient mechanical strength for supporting skin penetration, as well as to enhance the penetration with a relatively small base in the combination of the smoothly tapered profile^7,44^. Furthermore, the needle pitch of 300 μm enables mitigation of the bed-nail effect, while maintaining the shear stress distribution during skin penetration to minimize needle breakage. For the hole-side etching, the 30 μm-diameter holes are designed to be anisotropically etched to 300 μm deep (with an aspect ratio of 10) to provide sufficient overlap between the pillars and holes, which actually determines the opening location of the holes on the sidewalls of the sharpened needles. The borehole was intentionally shifted 30 μm from the center of the column to mitigate tissue coring and improve the sharpness.

It is also noteworthy that a single or small number of needles frequently have clogging issues, wherein the microneedle bore is blocked by skin tissue. As such, an adequately large number of microneedles is necessitated to maximize the ISF access volume and consistency, given the requirements for a shallow penetration depth. The two-dimensional array design is also desirable to accomplish a multiplexed sensing system within a single chip for the simultaneous and selective screening of target biomarkers in ISF.

## Micromachining process

Deep-reactive ion etching enables highly anisotropic silicon etching with high-selectivity relative to photoresists, making it feasible to fabricate structures with high-aspect ratios (i.e., the ratio of depth to width)^41^. However, the etching rate rapidly decreases with increasing aspect ratios of the structures being etched, which is termed aspect ratio-dependent etching (ARDE)^37,41,47^. In this case, the etching rate rapidly decreases with the aspect ratio of the etched structures. Above a certain critical point of the aspect ratio, the etching rate reaches a constant extremely low value^48^. The proposed mechanism lies in that the ion flux to the bottom of the structure decreases along with its aspect ratio, resulting in insufficient passivation layer removal^47^. As such, holes with higher aspect ratios would start to pinch off at the bottom, and above a certain critical point of the aspect ratio, the etching rate would reach a constant extremely low value. The plasma provides energetic species (bombarding positive ions) that are accelerated toward the wafer surface by a strong electric field. To mitigate the effects of ARDE, an independent depassivation step was inserted into the standard dual-phase Bosch process (i.e., passivation and etching)^49^. In this step, the energetic ions (e.g., argon) directionally bombard the bottom of the etched holes, which are conformally deposited with a Teflon-type polymer during the preceding passivation step. Such a triple-phase DRIE (i.e., passivation, depassivation, etching) can efficiently remove the passivating polymers, enabling anisotropic etching with very high-aspect ratios and without undesirable tapered sidewall profiles.

In our experiment, the anisotropic etching of silicon structures (i.e., pillars, holes) was conducted in an inductively coupled plasma reactive ion etcher (ICP-RIE). Specifically, the pillars were etched with a standard dual-phase Bosch DRIE recipe (i.e., passivation, etching) using an Oxford Instruments PlasmaLab 100 etcher. The holes were etched with a triple-phase modified Bosch recipe (i.e., passivation, depassivation, etching) using an Oxford Instruments PlasmaPro Estrelas100 etcher. The parameters of the “standard Bosch” recipe and the “modified Bosch” recipe are listed in Table 1. The DRIE system is configured for 4-inch wafers, and smaller samples (e.g., square shapes with side lengths of 3–5 cm) can be accommodated by mounting to a 4-inch silicon carrier wafer using Fomblin oil as a thermally conductive adhesive. The wafer is clamped with continuous helium backside cooling to ensure a constant wafer temperature, i.e., 15 °C for the “standard Bosch” and 5 °C for the “modified Bosch” processing.

**Table 1:** Parameters of the DRIE etching process with an RF frequency of 13.56 MHz

		ICP power (W)	Cycle (*s*)	Gas	Flow Rate (sccm)	RF power (*W*)	Pressure (mTorr)	Etching rate
Standard Bosch at 15 °C	Passivation	1000	5	C_4_F_8_	160	5	20	1.8 μm/min 0.3 μm/cycle
	Etching	1000	7	SF_6_	160	20	25	
Modified Bosch at 5 °C	Passivation	2500	0.6	C_4_F_8_	150	Off	60	7.8 μm/min 0.4 μm/cycle
	Depassivation	2000	0.7	SF_6_/Ar	200/30	100	25	
	Etching	2500	2	SF_6_	400	Off	60	

Note that the etching rate here was determined from the pillar structure etching with a large open area (~500 μm), and the etching rate can vary for different structures

The microneedle pattern was fabricated using standard photolithography techniques on a (100) silicon wafer, with the major process steps schematically illustrated in Fig. 1. The Si wafers were cleaned by immersion into hydrofluoric acid (HF: deionized water = 1:10) for 30 s, followed by deionized (DI) water rinsing, and nitrogen blow drying. First, a bilayer of AZ 4620 photoresist at a total thickness of ~24 μm was spun onto one side (termed as the ‘backside’’) of a 4-inch wafer (double-side polished, Fig. 1a). Afterwards, ultraviolet (UV) light exposure was carried out with a mask aligner (Karl Suss MA6) at a dosage of 1600 mJ/cm^2^, followed by immersing the exposed sample into a developer solution (AZ 400 K 4:1 diluted developer). The photolithography formed an array of holes in the photoresist of ~30 μm in diameter (Fig. 1a). Then, DRIE (i.e., the “modified Bosch” process) was performed to etch 300 μm-deep boreholes into the backside of the wafer, defining a high-aspect ratio (HAR) structure (~1:10, Fig. 1b). Note that at this moment, the other side of the wafer (termed as ‘frontside’’) was still flat. The DRIE process was halted before the boreholes were etched through the wafer to its frontside. Similar to the backside patterning, the frontside pillar pattern was defined (Fig. 1c) with alignment to the holes on the backside. The AZ 4620 photoresist was patterned to create cylindrical pillars aligned to the boreholes. This alignment also enabled accurate patterning of the holes such that their centers were offset from the needle axis. This offset was to address the tissue coring issue within the needle bore during insertion^7^. Pillars of ~300 μm in height and 100 μm in diameter were then etched by DRIE (Fig. 1d), making an overlap of 100 μm between the pillars and holes. At this point, the boreholes were still not exposed on the frontside. Essentially, they were still buried channels. Afterwards, using a mixed solution of hydrofluoric acid and nitric acid, the circular pillars were sharpened into conical needles, and the through-wafer holes were fully opened (Fig. 1e). This sharpening was realized by taking advantage of the isotropic etching nature of the chemical mixture, in which the etching rate decreases from the needle tip to the base^50^. Sharpening was also demonstrated by using the isotropic plasma dry etching and the combination of “wet” and “dry” isotropic etching. Holes were exposed on the sidewall of the needles, creating channels from the needles to the wafer’s backside (Fig. 1f).

## Results and discussion

### Pillar etching

**Fig. 2:**
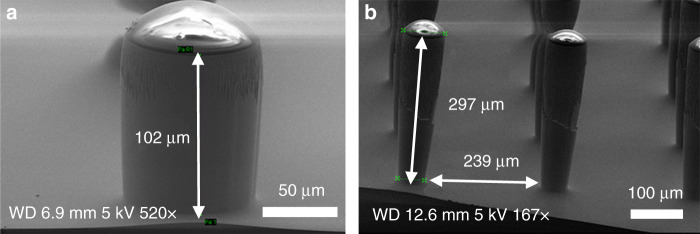
Pillar etching with **a** 300 cycles and **b** 900 cycles using the “standard Bosch” DRIE processing

The pillars were etched on a 4-inch Si wafer using dual-phase “standard Bosch” processing with the parameters shown in Table 1. The standard Bosch DRIE process was carried out for 300 cycles to etch 102 μm-high pillars with straight and smooth sidewall profiles (Fig. 2a). The pillar diameter was measured as 103 μm with negligible pattern erosion from the designed 105 μm, showing high-fidelity pattern replication. Upon extending the etching for another 600 cycles, the pillar height increased to 297 μm, whereas the pillar top decreased to 87 μm, which was caused by resistance erosion (Fig. 2b). The pillar base decreased to 55 μm, creating a reentrant (negatively tapered) profile (i.e., the top is wider than the base) as a result of ARDE on large open areas^51^. It is believed to be very challenging to control the profile at heights >100 μm for features with large gaps. This is because the plasma sheath starts to follow the etched features and results in some nonvertical ions and, hence, increased undercutting.

**Fig. 3:**
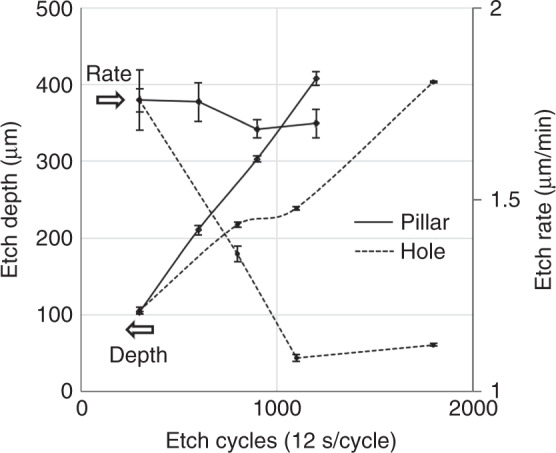
**The dependency of the etch depth and etch rate on the etch cycles using “standard Bosch” DRIE processing for the fabrication of pillars and holes**. The error bar represents the standard deviation from the average values

Figure 3 compares the dependency of the etch depth and etch rate on the etch cycles in the etching of pillars and holes using dual-phase “standard Bosch” processing. The error bar represents the standard deviation from the average values. The etch depth in the pillar etching linearly increases with the etch cycles, showing an almost constant etch rate of 1.8 μm/min. In contrast, the hole etching shows that the correlation between the etch depth and etch cycles gradually decreases (see supplementary Fig. 1), and hence, the etch rate decreases from 1.8 μm/min to 1.1 μm/min in the etching of deeper holes (>200 μm). The deceleration of the etching rate for high-aspect ratio structures is attributed to the ARDE. The physical ion bombardment during the etching step is insufficient to remove the fluoropolymers on the bottom of the hole structures^37^. Moreover, a positively tapered profile (opposite to the reentrant sidewall) was also observed in the etching of the 400 μm-deep holes, resulting from severe mask erosion (even with a double-layered photoresist) associated with the poor selectivity of the resist to Si (i.e., 1:18).

### Hole etching

**Fig. 4:**
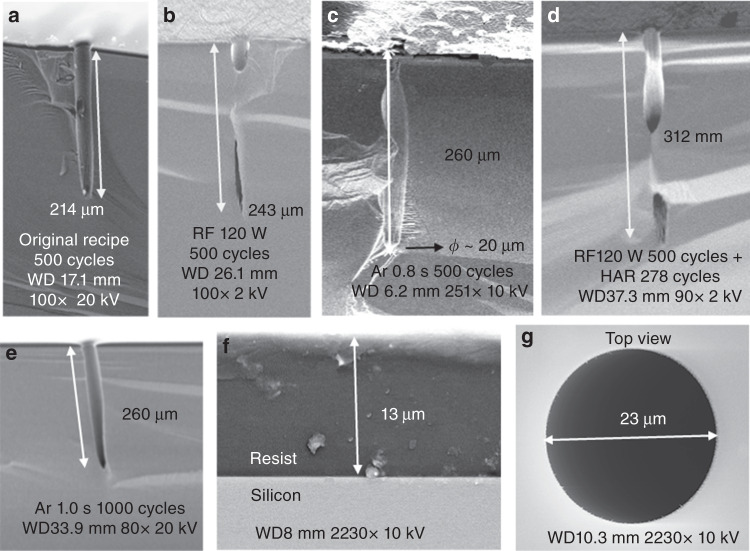
**The optimization of hole DRIE etching using triple-phase “modified Bosch” processing**

The dual-phase “standard Bosch” process consists of a passivation step and an etching step, suffering from ARDE for the etching of high-aspect ratio structures with well-controlled sidewall profiles. The triple-phase “modified Bosch” process has the distinctive feature of adding a depassivation step between the two steps of the standard Bosch. The depassivation step utilizes energetic Ar ions to efficiently remove the fluorocarbon passivating polymers (see details in Table 1). As such, the DRIE process provided the highly directional (almost completely anisotropic) etching of holes of 200 μm in depth and 30 μm in diameter. The etching mask on the wafer surface is faithfully replicated in the underlying silicon (Fig. 4a). The thicknesses of the photoresist (single layer) before and after the DRIE were respectively 10.65 μm and 9.10 μm. The selectivity of the photoresist with respect to Si was thus ~1:200, with a Si etching rate of 12 μm/min. Note that this etching also has the advantage of smaller scalloping due to the shorter etch cycle, resulting in a smoother surface that is beneficial to potential processing for the integration of sensing elements.

The etching of deeper holes (>200 μm) was also observed to decelerate as a result of insufficient passivation layer removal, similar to the case for etching with the dual-phase “standard Bosch” process. However, the triple-phase “modified Bosch” process enables adjusting the parameters of the independent depassivation (Ar ion bombardment) step for mitigating ARDE. To promote ion bombardment, the RF power (and hence, the acceleration voltage of the argon ions, Fig. 4b) and the Ar ion bombardment time (Fig. 4c) were respectively increased to enhance the removal of the passivating polymers at the bottoms of the holes (illustrated in Fig. 1b). As such, the etch depth increased without degrading the desired feature, especially upon using the combination of the original recipe and the HAR recipe (Fig. 4d). In another experiment, etching with the depassivation step of 1 s was carried out for 1000 cycles (Fig. 4e). The remaining photoresist was still sufficiently thick under the extended long ion bombardment (Fig. 4f), showing negligible pattern erosion (Fig. 4g). Note that the etching depth with a specific recipe has a standard deviation of <5 μm, showing high reproducibility.

### Needle sharpening

**Fig. 5:**
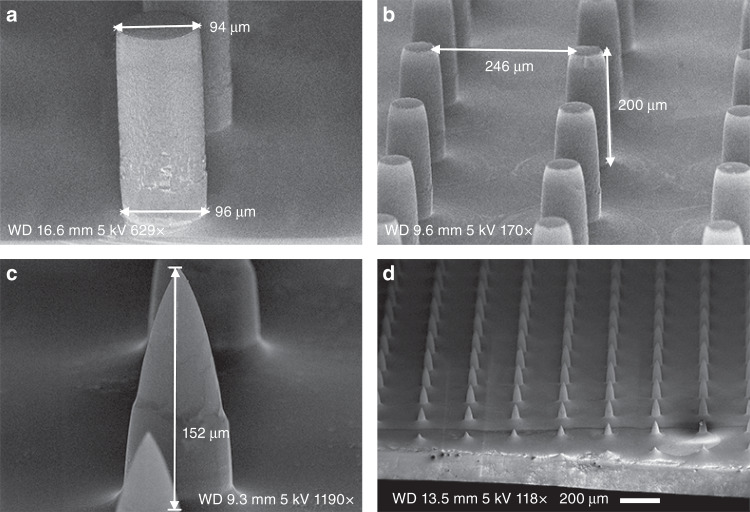
**The solid microneedle sharpening process using isotropic wet etching in a mixed solution of nitric acid and hydrofluoric acid**

The silicon micropillars were then sharpened into conical needles using an isotropic etching process (Fig. 5). This wet chemical etching of silicon utilizes a so-called HNA system, consisting of hydrofluoric acid (HF), nitric acid (HNO_3_), and a comparatively weak acetic acid (CH_3_COOH), which can be replaced with water^50,52^. The overall reaction involves the oxidation of silicon to SiO_2_ by HNO_3_ and subsequent SiO_2_ dissolution in HF. It is important that the overall reaction is limited by the diffusion of HF, and as a result, a ratio of 19:1 (which was adopted in this work) between HNO_3_ and HF was chosen to assure that the oxide formation dominates its removal. By placing the silicon micropillar sample at the bottom of a static solution, the HF diffusion to the silicon surface was made significantly slower than the dissolution reaction at the surface, and hence, it was the rate-limiting factor. HF reacts with SiO_2_ when they are in contact, rapidly consuming HF in the process. Owing to the large amount of exposed Si (and SiO_2_) between the pillars, the reactive species are significantly consumed at the pillar bottom, rather than at the pillar top. As the reaction proceeds in a static solution, the bottom has less replenishment than does the top, especially when the solution depth is well-controlled, which will result in faster etching (shrinking) of the pillar top. Another factor also contributes to the faster etch rate at the top—the fact that the edge is essentially exposed to HF from both vertical and lateral directions, whereas such etching will stop until a rounded (rather than sharp) shape is achieved.

In the isotropic wet etching experiment, square samples (1–2 cm side length) were used, and 600 cycles of “standard Bosch” DRIE etching was performed to fabricate solid micropillars. A 5 min wet etching resulted in a nearly vertical pillar of 94 μm wide and 217 μm high (Fig. 5a). Upon extending the etching time to 20 min, the pillar shrunk to 58 μm in diameter at the top and 200 μm in height (Fig. 5b), with the etching rate of appx. 1 μm/min in good agreement with the literature^50,52^. Upon adding another 20 min of etching, the blunt tip was sharpened into a single point of <1 μm, and simultaneously, the pillar height shrunk to 152 μm (Fig. 5c). By keeping the solution under static conditions (i.e., without agitation), as the etching proceeds, the etching species only diffuse from the bulk solution surrounding the silicon sample to replenish the solution contained within the spaces between the pillars. As a result, the sharpening shows high uniformity over the entire sample, with a percentage standard deviation of <5% for needle length (Fig. 5d), except in rows 1–3 on the sample edge, where the lateral diffusion from the adjacent open space becomes as important as the vertical diffusion (Fig. 5d). To achieve a higher degree of spatial uniformity across an entire wafer (including the edge), it is suggested to incorporate sacrificial structures (e.g., borders, extra rows of lines on the edge) to adjust the local concentration of etchant^50^.

### Simultaneous needle sharpening and hole opening

**Fig. 6:**
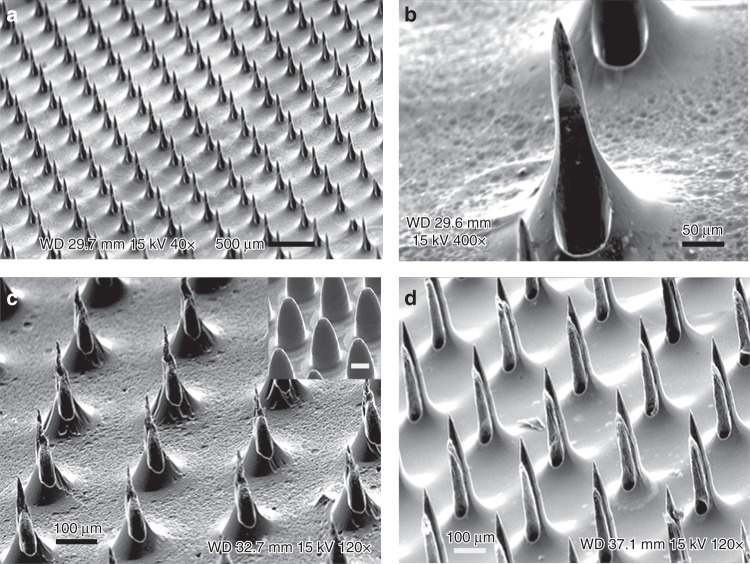
Hollow microneedle sharpening process using various methods. The hollow microneedle sharpening process using wet etching (**a**, **b**), a combination of wet etching and plasma etching (**c**), and plasma etching only (**d**). The inset in Fig. 6c shows the microneedle after wet etching and before plasma etching (scale bar 100 μm)

Afterwards, the micropillars incorporating holes were sharpened using a mixed solution of HNO_3_ and HF under stationary conditions. The isotropic etching—in particular, the etching from the lateral direction—simultaneously sharpened the needle tip and exposed the buried channel enclosed within the pillar. In such a manner, 160 μm-high hollow microneedles were fabricated after 40 min wet etching, with a percentage standard deviation of 2.7%, expressing high uniformity (Fig. 6a). Additionally, 30 μm-diameter holes were positioned off-center to mitigate the tissue coring issue during needle penetration, leading to so-called snake-fang needles^7^ (Fig. 6b). A similar shape was also fabricated by using the combination of isotropic “wet” and plasma “dry” etchings, with the needle height showing a percentage standard deviation of 1.5% (Fig. 6c). Here, the micropillars were etched in the HNA solution for 30 min to obtain a blunt tip (inset Fig. 6c), followed by a 15-min SF_6_ plasma etching in a reactive-ion etching system (Phantom II, Trion Technology Inc.). A SF_6_ plasma etching process alone for 35 min (without wet etching) with the PlasmaLab 100 etcher, in which the C_4_F_8_ gas was deactivated from the “standard Bosch” recipe, was also demonstrated to be capable of needle sharpening, with the needle height showing a percentage standard deviation of 1.3% (Fig. 6d). The aggressive plasma etching resulted in a nearly straight sidewall with a smoothly tapered profile and a narrow base to mitigate the incomplete needle penetration associated with skin elasticity^27,28^. Some of the major advantages of using plasma etching for needle sharpening include better process control and easy automation, as well as the elimination of handling strong oxidants.

### Capillary filling

**Fig. 7:**
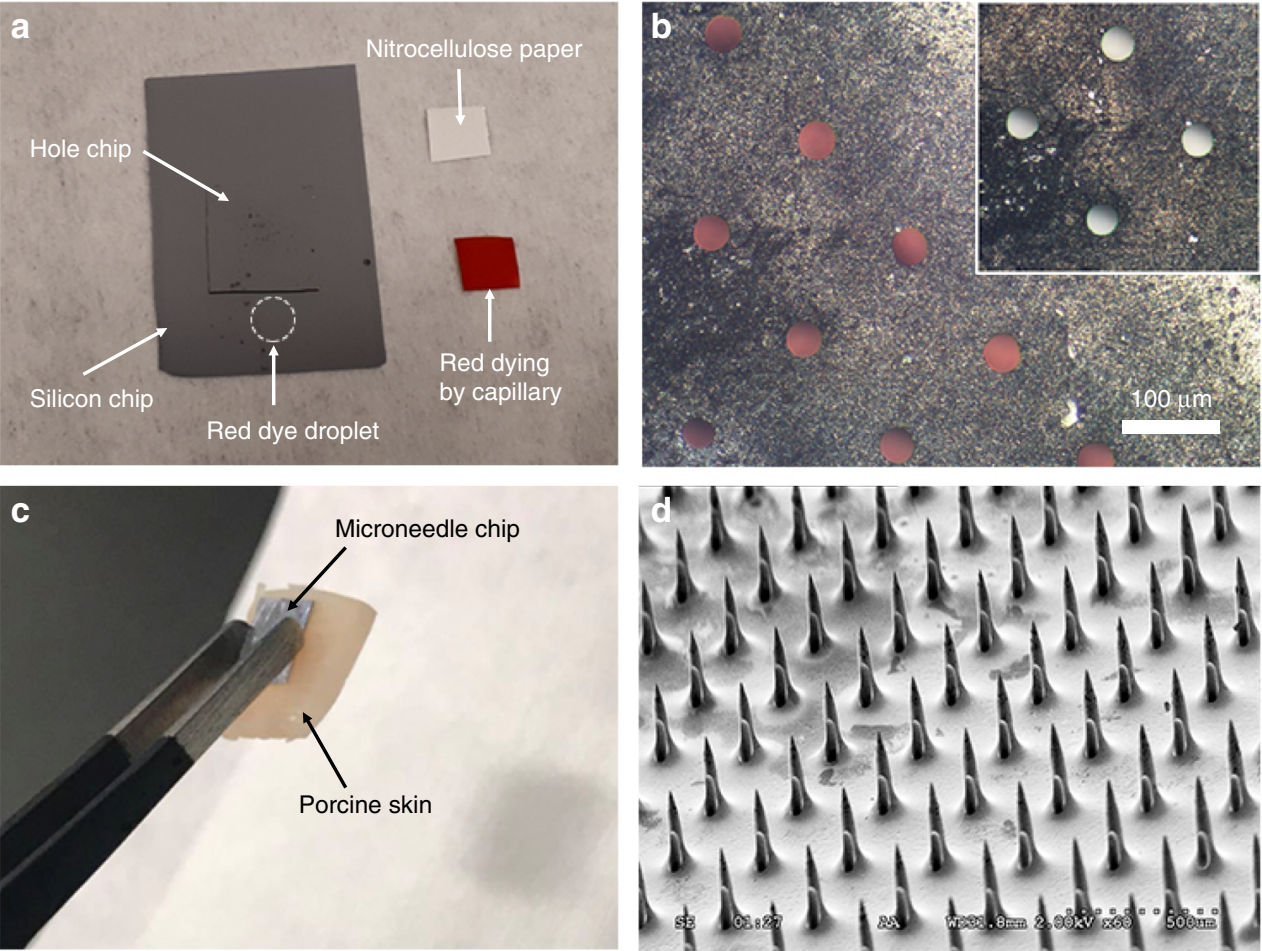
Capillary filling of DRIE-etched through-silicon holes and microneedle penetration into porcine skin. **a** Capillary filling of DRIE-etched through-silicon holes with a water solution of red dye and the microscopic images of holes **b** with capillary filling (inset: empty holes before filling). **c** Microneedle penetration into porcine skin; **d** microneedles remaining mechanically intact after repetitive penetrations

The capillary filling of DRIE-etched through-silicon holes (40 μm-diameter, without needle features) has been demonstrated using 1% weight/volume Allura red dye solution (0.1 g dye + 10 mL water, Fig. 7a). Oxygen plasma treatment is imperative to assure a hydrophilic surface with a water contact angle of <20 degrees^53^ by removing the passivating polymer (i.e., C_4_F_8_) left over from the previous Bosch DRIE process. The authors also observed that the lateral spreading of a water droplet was significantly larger on the Si surface after the oxygen plasma treatment. The sample with through-wafer holes was placed on the polished side of a clean silicon wafer (treated with the O_2_-plasma, Fig. 7a), creating a micrometer-level gap between the two surfaces to ensure effective capillary uptake. By placing a drop of the red dye solution in proximity to the hole chip (as indicated by the dashed circle in Fig. 7a), water uptake by the DRIE-etched through-wafer holes was accomplished in ~1–2 s, changing the color from white (empty holes, Fig. 7b inset) to red (Fig. 7b). The top surface with the polymer coating (without O_2_ plasma treatment) is hydrophobic, so the droplet on the top cannot spread out laterally. Upon placing a nitrocellulose paper on the top of the hole chip with a gentle finger push, the nitrocellulose paper was able to wick liquid through its capillary structure (Fig. 7a). This result shows the feasibility of direct integration between Si microneedles and paper microfluidics for the purpose of lateral-flow assay assembly.

### Skin penetration

Afterwards, the skin penetrability was assessed with excised porcine skin^54^. Porcine skin is often used as a model of human skin due to the similarities in anatomy such as the thickness of the stratum corneum. The insertion force is suggested to range from 0.1 to 3 N, such that a thumb-push is sufficient for skin penetration, rather than requiring aid from impact-insertion applicators^7,28^. A razor was used to remove the subcutaneous fat on the back of the skin, making 3–4 mm-thick porcine skin samples. Then, the skin with good surface conditions (without hair or skin disorders such as scarring) was cut into square pieces (2–3 cm length) for experimentation. Following IPA soaking, the microneedle chip was gently thumb-pushed onto the porcine skin sample for a few seconds, and the needles were capable of holding the skin sample (Fig. 7c), indicating successful skin penetration. The successful skin penetration is largely attributed to the extreme sharpness of the microneedles, as the insertion force linearly decreases with the interfacial area of the needle tip, according to a study^28^. Following separation from the skin, the microneedle chip was baked at 250 °C for 30 min on a hotplate for disinfection and sterilization by a burning (oxidation) process, while keeping the residual body tissues adsorbed onto the needle shank during skin penetration. This step also dried the sample to avoid contaminating the SEM chamber. Afterward, we carried out an scanning electron microscopy (SEM) inspection, in which we did not observe any mechanical failures. As such, the microneedles were concluded to be mechanically intact after repetitive skin penetration (Fig. 7d). This agrees well with the theoretical prediction regarding similar Si structures, in which the Si pillar fracture force was an order of magnitude greater than the insertion force necessary for skin penetration^28,44^. Note that the black-colored substances on the needle shanks are likely the burning products of residual body tissues.

## Conclusion

A DRIE process for fabricating silicon hollow microneedle arrays has been presented, aiming to explore the feasibility of microneedle-enabled in-vivo biosensors, in which the sensing elements can be incorporated on the inner surface the boreholes. The insertion of a depassivation process using directional Ar ion bombardment into the two standard Bosch half-cycles resulted in efficient removal of the passivating fluorocarbon layer, enabling highly anisotropic etching of circular holes with diameters as small as 30 μm to a depth of >300 μm. The needle tips were sharpened to single points with radii as small as 5 μm using either wet or dry plasma etching or the combination of both. This isotropic etching step also opened the holes originally embedded within the pillars. Such sharp microneedles have been demonstrated to be sufficiently robust to penetrate porcine skin without needing a mechanical applicator, with the needles remaining mechanically intact after repetitive penetrations. Capillary filling of the DRIE-etched through-silicon holes has also been demonstrated, showing their feasibility for transporting biomarkers of clinical interest to the sensing sites using capillary action.

## Supplementary information

Supplementary Material

Supplementary Information
