# Supplementary material for: Fabrication of sharp silicon hollow microneedles by deep-reactive ion etching towards minimally invasive diagnostics
Source: Microsyst Nanoeng. 2019 Aug 26;5:41. doi: 10.1038/s41378-019-0077-y (PMC6799813; doi:10.1038/s41378-019-0077-y)
Supplement: Supplementary file 1 — Supplementary Material [file 41378_2019_77_MOESM1_ESM.docx]

Fabrication of Sharp Silicon Hollow Microneedles by Deep Reactive Ion Etching Towards Minimally-Invasive Diagnostics

Yan Li^1,2,§^, Hang Zhang^1,§^, Ruifeng Yang^1^, Yohan Laffitte^2^, Ulises Schmill^2^, Wenhan Hu^1^, Moufeed Kaddoura^2^, Eric J. M. Blondeel^2,*^, and Bo Cui^1,*^

^1^Department of Electrical and Computer Engineering, University of Waterloo, 200 University Ave. West, Waterloo, ON, N2L 3G1, Canada

^2^ExVivo Labs Inc., 3 Regina St. N, Waterloo, ON, N2J 2Z7, Canada

**§: Both authors contributed equally to this manuscript.**

***: To whom correspondence should be addressed. Email: eric@exvivo.com,** [**bcui@uwaterloo.ca**](mailto:bcui@uwaterloo.ca)


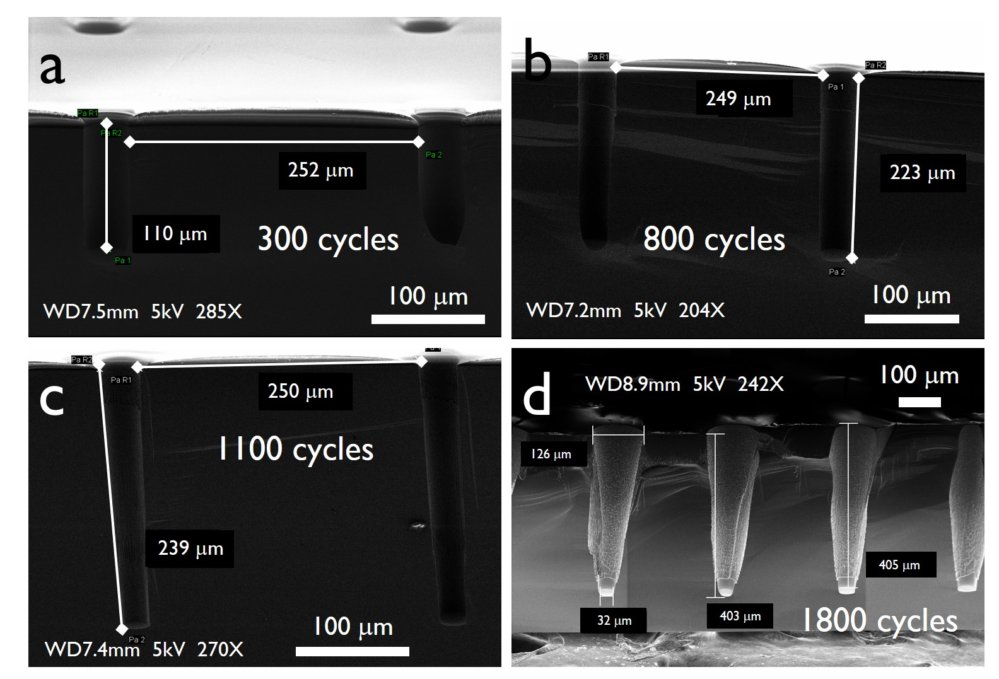


**Supplementary Figure 1** Hole etching with (a) 300 cycles, (b) 800 cycles, (c) 1100 cycles and (d) 1800 cycles using the “Standard Bosch” DRIE processing.

The dual-phase “Standard Bosch” processing was utilized for the hole etching. The standard Bosch DRIE process was carried out for 300 cycles to etch 110 μm deep holes with a diameter of 40 μm, faithfully replicating the design; the sidewall profile was straight and smooth (Supplementary Fig. 1a). The remaining photoresist (double-layered) was approx. 18 μm thick and hence 6 μm was etched away in the DRIE step, giving a selectivity of 18 relative to the silicon etching. Adding another 500 cycles etching resulted in 225 μm deep holes, with a flat bottom indicating efficient removal of Teflon-type polymer on the hole bottom (Supplementary Fig. 1b). Extending another 300 cycles (i.e., 1100 cycles in total) etching slightly increased the hole depth to 240 μm, but the bottom shrank to 24 μm from 40 μm in design (Supplementary Fig. 1c). Extending another 700 cycles (i.e., 1800 cycles in total) etching resulted in approx. 400 μm deep holes; the top of holes had a diameter of 126 μm and the bottom of 31 μm (Supplementary Fig. 1d).
