# Supplementary material for: Fabrication of sharp silicon hollow microneedles by deep-reactive ion etching towards minimally invasive diagnostics
Source: Microsyst Nanoeng. 2019 Aug 26;5:41. doi: 10.1038/s41378-019-0077-y (PMC6799813; doi:10.1038/s41378-019-0077-y)
Supplement: Supplementary file 2 — Supplementary Information [file 41378_2019_77_MOESM2_ESM.docx]

Advanced Deep Reactive Ion Etching of Silicon Hollow Microneedles for Transdermal Interstitial Fluid Extraction

Hang Zhang^1,§^, Yan Li^1,2,§^, Ruifeng Yang^1^, Yohan Laffitte^2^, Ulises Schmill^2^, Wenhan Hu^1^, Moufeed Kaddoura^2^, Eric J. M. Blondeel^2,*^, and Bo Cui^1,*^

^1^Department of Electrical and Computer Engineering, University of Waterloo, 200 University Ave. West, Waterloo, ON, N2L 3G1, Canada

^2^ExVivo Labs Inc., 3 Regina St. N, Waterloo, ON, N2J 2Z7, Canada

**§: Both authors contributed equally to this manuscript.**

***: To whom correspondence should be addressed. Email: eric@exvivo.com,** **bcui@uwaterloo.ca**


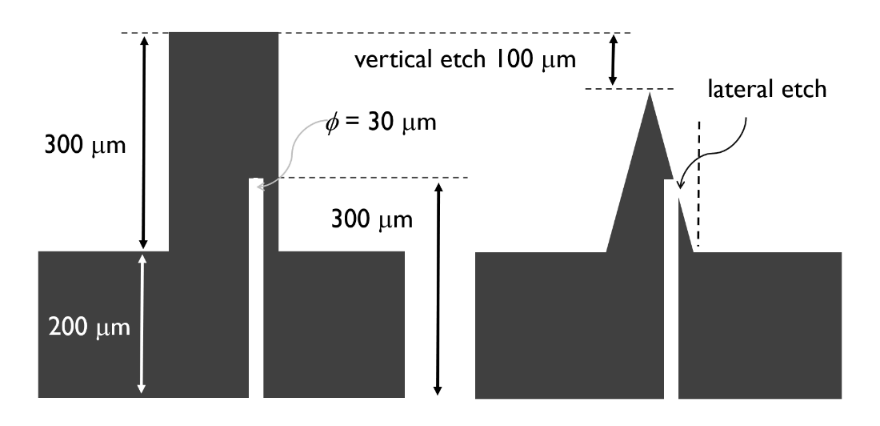


Supplementary Figure 1 Schematic diagram of the processing to fabricate a silicon hollow microneedle: the deep hole (i.e., 300 μm) anisotropic etching with high aspect ratio (i.e., 10), and the simultaneous needle sharpening and hole exposing by taking advantage of the isotropic nature of the wet etching.


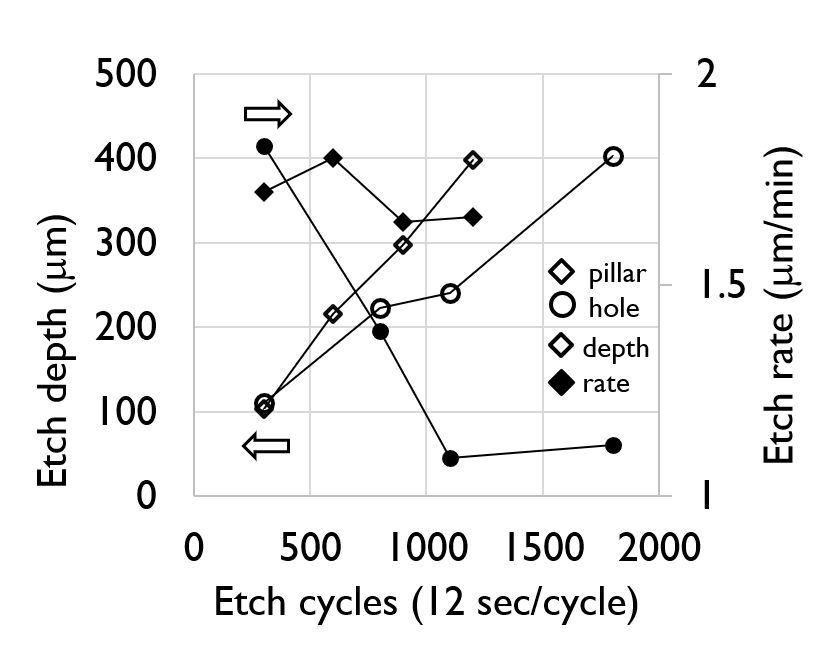


**Supplementary Figure 2** The pillar and hole etch dependent on etch cycles using the “Standard Bosch” DRIE processing.
